# Clinical, Cytologic, Histopathologic, and Diagnostic Imaging of a Malignant Peripheral Nerve Sheath Tumor in the Renal Pelvis of a Border Collie Dog

**DOI:** 10.1111/vcp.70077

**Published:** 2026-01-15

**Authors:** Félix Romero‐Vélez, Bárbara Serrano, Javier Martínez‐Caro, Sonia González‐Rellán, Rosa Novellas, Alicia García‐Ferrer, Josep Pastor, Laia Solano‐Gallego

**Affiliations:** ^1^ Hospital Clínic Veterinari, Universitat Autònoma de Barcelona Bellaterra Spain; ^2^ Servei de Diagnòstic de Patologia Veterinària Universitat Autònoma de Barcelona Bellaterra Spain; ^3^ Servei D'Hematologia Clínica Veterinària Universitat Autònoma de Barcelona Bellaterra Spain; ^4^ Departament de Medicina i Cirurgia Animal, Facultat de Veterinària Universitat Autònoma de Barcelona Bellaterra Spain

**Keywords:** canine, hematuria, hypertrophic osteopathy, immunohistochemistry, kidney

## Abstract

A 12‐year‐old female spayed Border Collie dog was presented for evaluation of 6 months of intermittent hematuria and weight loss. A highly vascularized right renal mass deforming the renal architecture and paraneoplastic hypertrophic osteopathy were found. Cytologic evaluation of the mass obtained by fine‐needle aspiration guided by ultrasound revealed mesenchymal cells with a moderate amount of bluish cytoplasm, moderately defined cell borders, and spindle to stellate or roundish morphology. The nuclei were centrally located, with a coarse chromatin pattern, round to oval, and occasionally bean‐shaped. Usually, a single distinct nucleolus per nucleus with minimal size variation was noted. Anisocytosis and anisokaryosis were moderate. The cytologic interpretation was mesenchymal proliferation with moderate atypia, most consistent with soft tissue sarcoma. Right ureteronephrectomy was performed. Histologic evaluation showed a neoplastic proliferation located beneath the lamina propria of the transitional epithelium of the renal pelvis and infiltrating the renal medulla. Immunohistochemistry for protein S‐100, laminin, and desmin was performed to further characterize the lesion as a nerve sheath tumor. The hematuria disappeared 4 days after the surgery; 5 months later, no alterations were observed in the general examination, and the paraneoplastic hypertrophic osteopathy mildly improved. This is the first cytologic description of a primary renal malignant nerve sheath tumor in the renal pelvis of a dog with paraneoplastic hypertrophic osteopathy.

## 1 Case Presentation

A 12‐year‐old female spayed Border Collie dog was presented for evaluation of intermittent hematuria and weight loss of 6 months duration. On presentation, a body condition score of 3/9 was observed; no further alterations were detected during the physical examination. A complete blood cell count (CBC) was performed on the Sysmex XN‐1000 V (Sysmex Corporation, Norderstedt, Germany), showing a moderate normocytic normochromic non‐regenerative anemia (hematocrit 25%, reference interval: 37%–55%). A serum biochemistry profile was performed on the chemistry analyzer AU480 (Beckman Coulter, California, USA), showing no abnormalities. A urinalysis was performed on a sample obtained by cystocentesis; biochemical analysis of the urine using Combur^10^ Test strips (Roche) revealed proteinuria (4+, reference interval: Negative—Trace) and heme (4+, reference interval: Negative). The urine specific gravity (USG) was 1.031, and the sediment confirmed hematuria with more than 100 red blood cells (RBC) per high‐power field (HPF). Urine was submitted for bacterial culture, and no bacteria were isolated.

**FIGURE 1:**
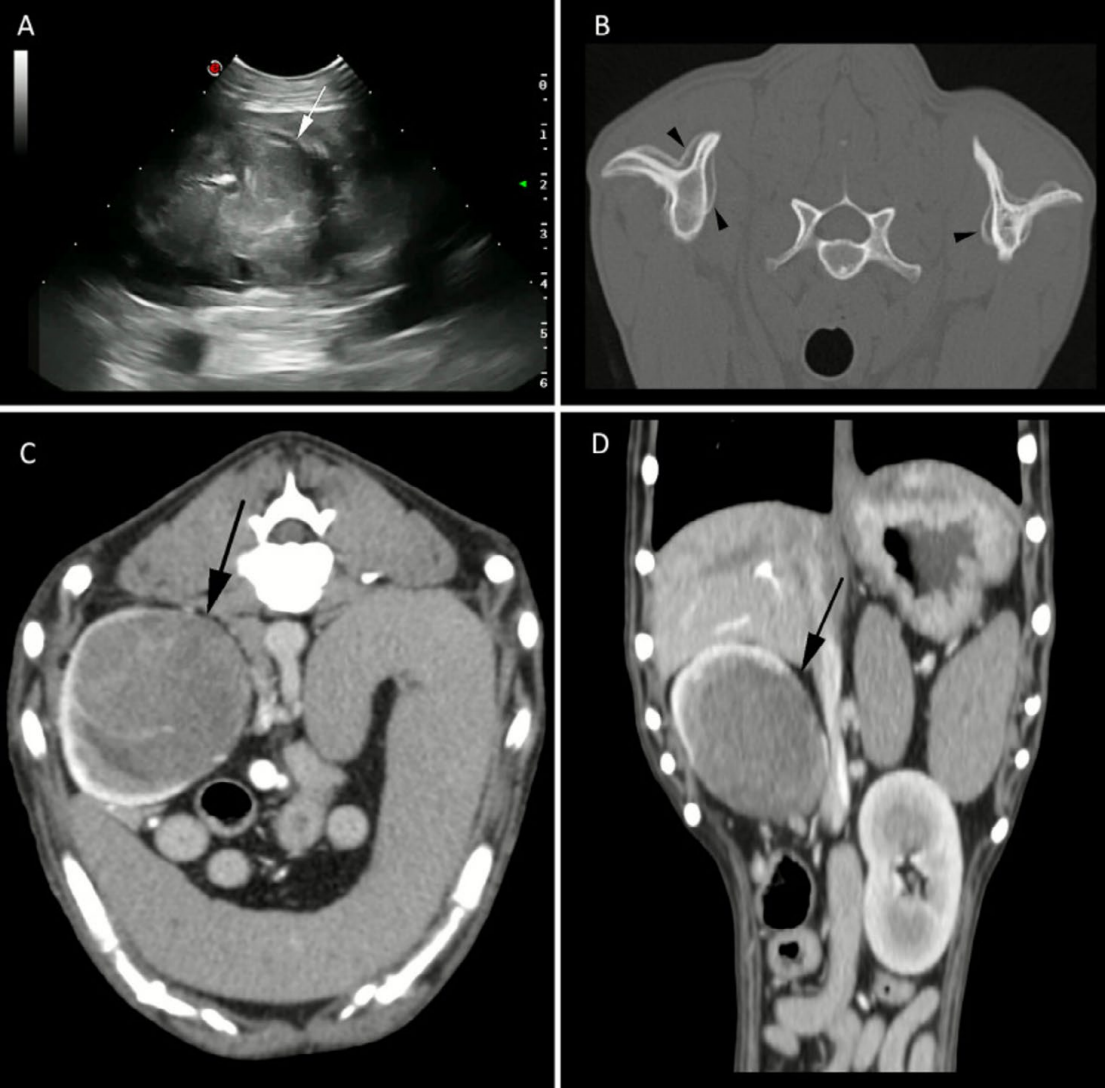
Diagnostic images of the right renal mass from a 12‐year‐old female spayed Border Collie dog. (A) Ultrasonographic image of the mass (white arrow) appearing as a heterogeneous and poorly defined structure, with irregular margins, that deforms the renal architecture. (B) Computed tomographic image showing a periosteal reaction of the scapulae (black arrowheads) consistent with hypertrophic osteopathy at the time of diagnosis. (C, D) computed tomographic images of the renal mass (black arrow) in soft tissue algorithm, after intravenous contrast administration. The mass shows heterogeneous and mild contrast enhancement and poorly defined margins.

Abdominal ultrasound revealed a highly vascularized right renal mass deforming the renal architecture, with loss of visualization of the normal renal cortex and medulla, as well as the margins of the pelvis (Figure 1A). A full‐body computed tomographic (CT) study showed a 5.1 × 4.5 cm oval right renal mass with poorly defined margins (Figure 1C,D). It was heterogeneously hypoattenuating and, after intravenous contrast administration, it showed mild enhancement, most marked on the late venous phase. The caudal segment of the right ureter was mildly distended. A diffuse and lamellar periosteal reaction was detected on the CT scan (Figure 1B), extending through the long bones of the four limbs, from the phalanges to the scapulae and pelvis.

Results of the CBC, serum biochemistry, urinalysis, abdominal ultrasound and CT suggested a neoplastic renal mass without evidence of metastasis and paraneoplastic hypertrophic osteopathy (PHO), and adequate renal function with chronic non‐regenerative anemia secondary to urinary tract neoplasia, although hematuria could be contributing to the anemia.

**FIGURE 2:**
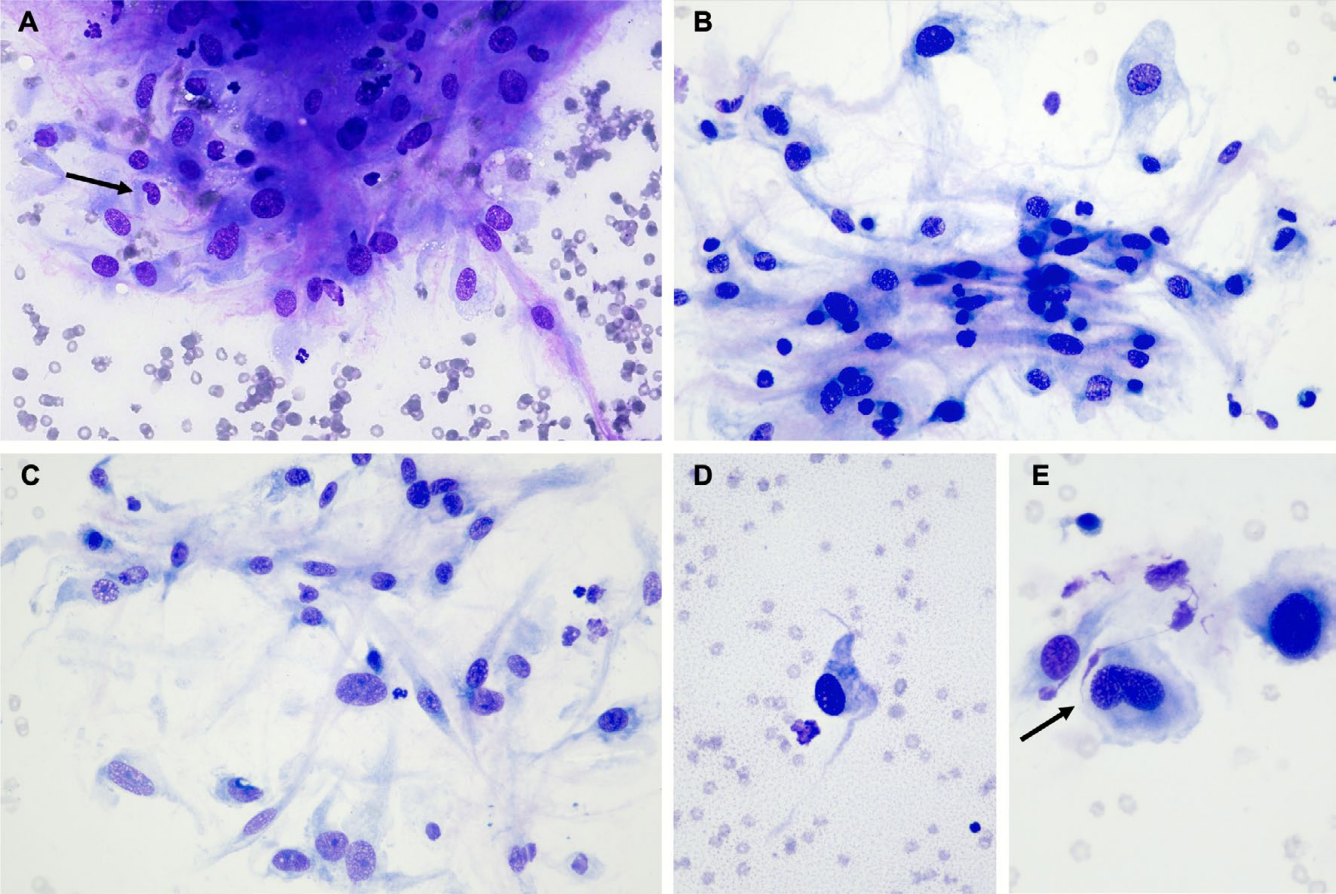
Cytologic micrographs of the fine needle aspirate of the right renal mass of a 12‐year‐old female spayed Border Collie dog, showing a mesenchymal cell population, with moderate anisokaryosis and anisocytosis. Sporadic bean‐shaped nuclei were present, as shown in images A and E (arrows). Quick aqueous Romanowsky stain, original magnification ×40 (A), ×40 (B), ×40 (C), ×40 (D), and ×60 (E), objectives.

An ultrasound‐guided fine needle aspiration of the right renal mass was performed, and slides were stained with a quick aqueous Romanowsky stain (Panóptico Rápido, Química Clínica Aplicada S.A., Amposta, Spain) (Figure 2). There was a moderate number of mesenchymal cells, distributed either individually or forming loose aggregates with a storiform distribution. The cells had a moderate amount of bluish cytoplasm, moderately defined cell borders, and spindle to stellate or roundish morphology. Nuclei were centrally located, round to oval, occasionally bean‐shaped (Figure 2E), with a coarse chromatin pattern and frequently, a single, distinct, round nucleolus, with minimal size variation. Moderate anisocytosis and anisokaryosis, and low to moderate nuclear to cytoplasmic ratios were observed. Variable amounts of pink, fibrillar extracellular matrix material were noted admixed with the mesenchymal cells. In some areas, there was a pink, granular proteinaceous background (Figure 2D). There were also a significant number of activated macrophages and a few non‐degenerate neutrophils. Glomeruli and/or tubular epithelial cells were also encountered sporadically. The primary cytologic interpretation was mesenchymal proliferation with moderate atypia, most consistent with a soft tissue sarcoma. However, while a reactive mesenchymal proliferation (i.e., fibroplasia, granulation tissue) was considered a differential diagnosis, it was thought to be less likely. A laparotomy by midline and paracostal approach under general anesthesia was performed to remove the renal mass. The mass was causing enlargement of the right kidney, with invasion and distension of the right renal vein. Inflammation of the perirenal adipose tissue and neovascularization were visible intraoperatively. A unilateral right ureteronephrectomy was performed. A diffuse incisional catheter 16Ga × 30 cm (MILA International Inc. USA) in the subcutaneous tissue was placed for postoperative pain control with Ropivacaine 0.2% instillation. The patient was discharged after 48 h with no postsurgical complications.

**FIGURE 3:**
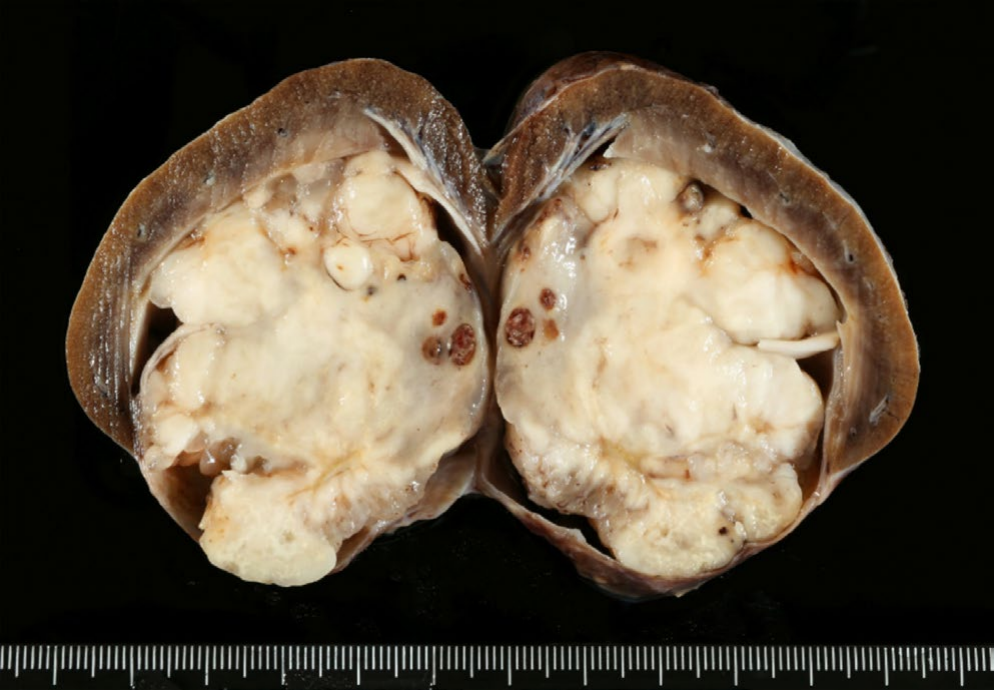
Gross pathology of the right kidney from a 12‐year‐old female spayed Border Collie dog. A longitudinal section revealed a multilobulated, firm, white‐to‐tan nodule with a diameter of 4 cm, which was occupying the renal pelvis and compressing the renal parenchyma.

**FIGURE 4:**
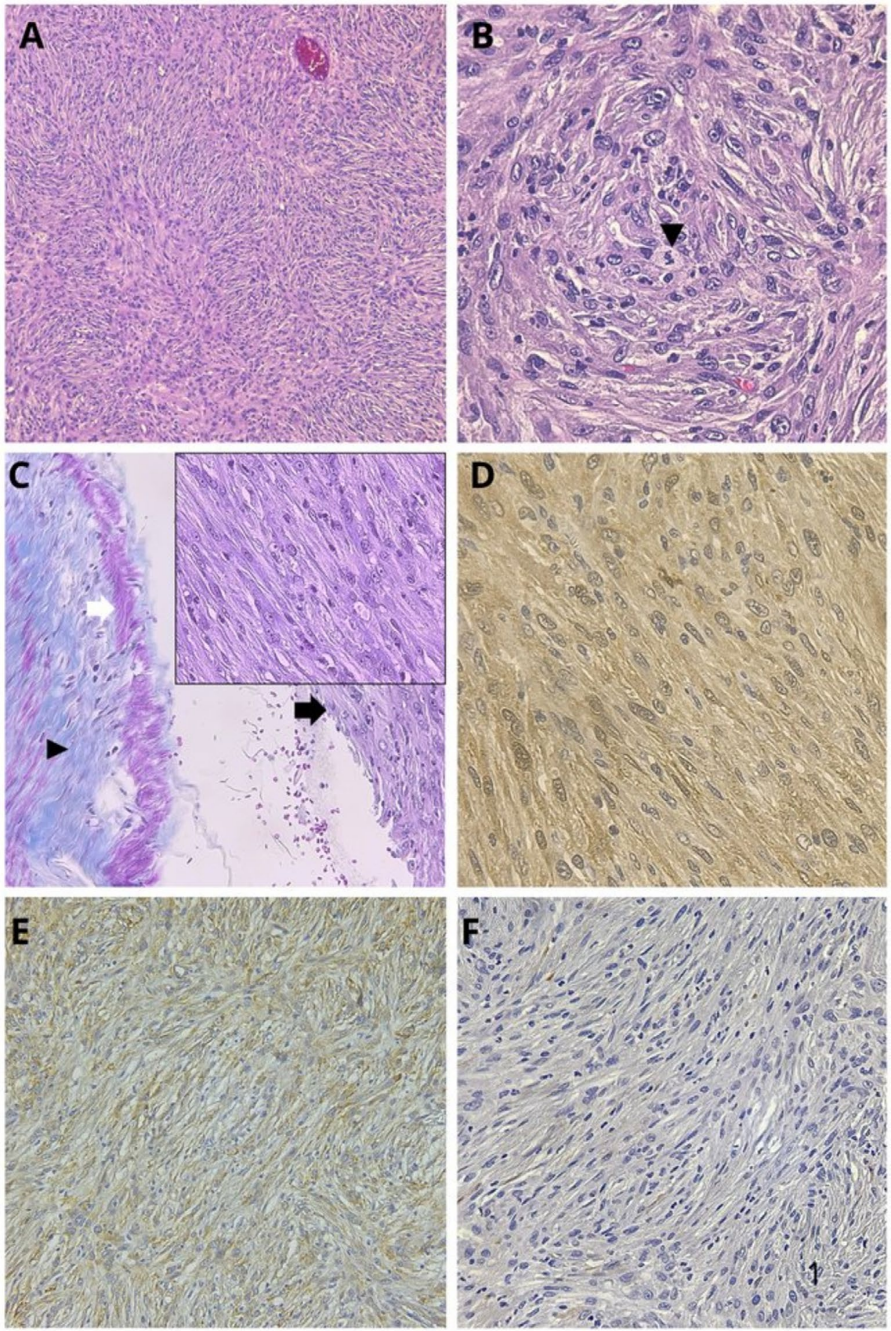
Histologic and immunohistochemical examination of the right renal mass from a 12‐year‐old female spayed Border Collie dog. At low magnification, the mass appeared as a densely cellular neoplastic proliferation composed of spindle cells arranged in bundles, interlacing streams, and whorls (A). At higher magnification, the neoplastic cells were large, spindle‐shaped to pleomorphic, with indistinct cytoplasmic borders, prominent nuclei, and occasional aberrant mitotic figures (black arrowhead) (B). Masson's trichrome staining of the renal mass revealed diffuse and homogeneous blue‐pink staining of the neoplastic cells (black arrow and inset), pink‐red staining in the smooth muscle of the arterial tunica media (white arrow), and blue staining in the elastin of the same arterial layer (arrowhead), the inset show the diffuse blue‐pink staining of the neoplastic cells (C). S100 immunohistochemistry demonstrated diffuse cytoplasmic positivity in approximately 80% of the neoplastic cells with rare nuclear staining (D). Laminin immunohistochemistry showed strong cytoplasmic positivity in the neoplastic cell population with rare nuclear staining (E). Desmin immunohistochemistry was negative in the neoplastic population (F). Hematoxylin and eosin, 10× objective (A) and 60× objective (B); Masson's trichrome, 20× objective, 60× inset (C); S100 immunohistochemistry, 60× objective (D); laminin immunohistochemistry, 20× objective (E); desmin immunohistochemistry, 40× objective (F).

The right kidney was submitted for histologic examination. The kidney was longitudinally sectioned, and the renal pelvis was completely effaced by a spherical, white, finely mottled, solid, and firm nodule measuring 4 × 5 cm that compressed the medulla and cortex of the kidney (Figure 3). A section of the kidney containing the mass was sampled, fixed for 24 h by immersion in 10% neutral‐buffered formalin, paraffin wax embedded, 4 μm sectioned, and stained with hematoxylin and eosin. Histologically, the mass consisted of a non‐encapsulated, poorly circumscribed, densely cellular neoplastic proliferation located beneath the lamina propria of the transitional epithelium of the renal pelvis and infiltrating the renal medulla. Cells were mainly arranged in dense streams, fascicles, and whorls with sparse collagenous stroma (Figure 4). Neoplastic cells were fusiform to pleomorphic with abundant light eosinophilic cytoplasm, occasionally fibrillary, indistinct cytoplasmic borders, and basophilic oval nuclei. The mitotic count was low (1–2 mitotic figures/10 HPF). Anisocytosis and anisokaryosis were marked, and megalocytosis, multinucleated cells, and multifocally karyorrhectic and karyolytic debris were observed (Figure 4). The cortex and medulla of the affected kidney parenchyma were markedly reduced. The remaining parenchyma showed moderate to severe diffuse fibrous deposition within the interstitium and loss of a high number of tubules and glomeruli, indicating severe atrophy. Standard Masson's trichrome staining was performed and showed a diffuse cytoplasmic and nuclear blue‐pink staining in the neoplastic population, pink‐red in the smooth muscle of the arterial tunica media, and blue in the elastin artery layer adjacent to the tumor. Based on the growth pattern characterized by bundles and whorls, along with the absence of a distinct stromal matrix—evidenced by the lack of both blue and pink‐red staining on Masson's trichrome—and the presence of malignant features, a presumptive diagnosis of malignant peripheral nerve sheath tumor (MPNST) was established. To confirm the diagnosis, immunohistochemistry for both S‐100 [1, 2] (using a polyclonal rabbit anti‐S100 antibody [Dako], followed by detection with Master Polymer Plus [which includes an HRP‐conjugated secondary system] and DAB as the chromogen) and laminin [1, 2] (using a polyclonal rabbit anti‐laminin antibody [Dako], followed by detection with the NeoStain Poly 1‐Step Kit [Neo Biotech] without chromogen, with DAB applied separately as the chromogen) was performed (Figure 4D,E, respectively). Both markers showed positive, finely granular cytoplasmic staining (Figure 4D,E) and were considered to be positive. Desmin was also performed (using monoclonal mouse Anti‐human Desmin Clone, Dako, as primary and master polymer Plus HRP of Vitro, as secondary) to rule out a muscular tumor, but staining was negative (Figure 4F). The final histologic diagnosis was malignant peripheral nerve sheath tumor (MPNST) in the renal pelvis.

The hematuria disappeared 4 days after the surgery. In the follow‐up evaluations at 12 days, 1 month, and 5 months, the dog had gained weight, and no abnormalities were observed in the physical examination. The 5‐month follow‐up included a full‐body CT, which revealed the absence of the kidney and ureter with no evidence of metastatic disease, and a less severe periosteal reaction in the long bones. These changes suggested that the PHO mildly improved after the nephrectomy. At this 5‐month recheck, a CBC, biochemistry profile, and urinalysis were performed, and all results were within normal limits.

## 2 Discussion

Nerve sheath tumors (NSTs) are a group of neoplasms that arise from Schwann cells, perineurial cells, and epineurial or endoneurial fibroblasts [3]. In human medicine, reports of NSTs arising in the kidney are rare [4, 5]. In dogs, most MPNSTs occur in the paraspinal, peripheral, and spinal nerves; they may also occur in other uncommon sites such as the liver, spleen, adrenal gland, urinary bladder, diaphragm, and tongue [6]. In small animals, there is one case report of a malignant renal schwannoma in a cat [7] and, most recently, one MPNST in the kidney of a dog [6].

In human medicine, renal schwannomas are usually located in the renal hilum or pelvis; it is believed that this is because the main nerves of the kidney accompany the renal artery and enter at the renal hilum [5]. In an MPNST case reported in a dog, the tumor was detected at the caudal pole of the left kidney [6]. In the case described here, the tumor originated in the renal pelvis, in agreement with the anatomic localization described in human medicine [5].

The cytologic examination of this case was most consistent with a soft tissue sarcoma. Differential diagnoses for these cytologic findings in a canine renal mass included primary renal sarcoma, MPNST, leiomyosarcoma, hemangioma, hemangiosarcoma, undifferentiated pleomorphic sarcoma (previously malignant fibrous histiocytoma), fibroleiomyosarcoma, or metastatic soft tissue sarcoma of other locations, among others [8]. Cytologic descriptions of canine MPNSTs are scant within the veterinary literature [9]. In general, cytomorphology is not pathognomonic, and significant overlap exists between MPNST and other soft tissue sarcomas. The main cytologic features that support a neural origin include a spindle cell proliferation with slender ovoid, comma‐shaped, or bean‐shaped nuclei [10, 11]. It is noteworthy that sporadic bean‐shaped nuclei were observed in the present case (Figure 2E). Some cases may show pink, fibrillar extracellular matrix or collagenous stroma associated with the neoplastic cell population [10], as in the present study. The proteinaceous, pinkish, granulated background noticed in some areas of our cytologic slides has also been described in other reports [9]. No significant cytoplasmic vacuolation was present in our case. An epithelioid MPNST variant has also been described and typically shows more cellular pleomorphism [10].

A definitive diagnosis of an MPNST is not possible based on cytology alone. However, this technique is inexpensive, easy to perform under sedation, and can be part of the diagnostic protocol, complementing other tests such as diagnostic imaging, histopathology, and immunohistochemical staining evaluation. Histologically, the characteristic pattern arrangement in whorls, bundles, and streams with the absence of abundant stroma suggested a nerve sheath tumor with malignant features (infiltrative nature, hypercellularity, mitotic count, marked anisocytosis and anisokaryosis). The differential diagnosis based on histological examination included other soft‐tissue sarcomas, such as skeletal or smooth‐muscle tumors and fibrosarcomas, which can be difficult to differentiate on histologic examination alone. Masson's trichrome showed a diffuse cytoplasmic and nuclear blue‐pink staining in the neoplastic population, in contrast to the pink‐red staining typically associated with muscular neoplasms and the blue staining expected in fibrosarcomas [2]. Immunohistochemistry markers used to identify cells of nerve sheath origin in veterinary species include S100, Glial Fibrillary Acidic Protein (GFAP), Nerve Growth Factor receptor (NGFR), Neuron Specific Enolase (NSE), laminin, and CNPase [2]. In the current case, S100 and laminin were performed with a strong diffuse positive reaction. S100 is a protein, considered a calcium flux regulator, expressed in cells of neural crest origin and used in the diagnosis of NST [2]. Laminin immunohistochemistry is often employed for diagnosing NSTs. Schwann and perineurial cells generate a significant amount of basal lamina proteins that react with laminin and type IV collagen with immunohistochemical stains [1]. Desmin was also performed to rule out a muscular tumor, but staining was negative (Figure 4F). A definitive diagnosis of malignant peripheral nerve sheath tumor of the renal pelvis was made based on the clinical, cytologic, histologic, and immunohistochemical evaluation.

Clinical signs of MPNSTs are related to the site of the tumor, most commonly proprioceptive deficits, lameness, muscle atrophy, diminished muscular tone, or cranial nerve alterations [9, 12]. In this case, chronic hematuria and weight loss were the only signs reported at presentation. In dogs, PHO is most frequently diagnosed in association with primary or metastatic pulmonary neoplasia [13]. However, other reports describe PHO secondary to infectious/inflammatory lung disease, *Dirofilaria immitis* infection, *Spirocera lupi* esophageal granuloma, bacterial endocarditis, right‐to‐left shunting with a patent ductus arteriosus, esophageal foreign body or congenital megaesophagus, and extrapulmonary neoplastic causes, including tumors of the bladder and kidney [13]. Metastasis is rare in MPNSTs [14]. The initial CT in this case revealed no evidence of metastasis, but the long bone changes were consistent with PHO. A follow‐up CT performed 5 months later showed no metastasis and a decrease in the periosteal reaction of the four limbs, which was smooth and well‐defined, further confirming the paraneoplastic origin of the PHO.

In conclusion, the findings described in the present case may be considered in the future for the differential diagnosis of renal masses in canine patients. To the best of our knowledge, this is the first cytologic description of primary renal MPNST in the renal pelvis of a dog with PHO.

## Conflicts of Interest

The authors declare no conflicts of interest.
